# Comment to the article: “Consensus statement on the definition of orthostatic hypertension endorsed by the American Autonomic Society and the Japanese Society of Hypertension” by Jordan and colleagues

**DOI:** 10.1007/s10286-022-00913-x

**Published:** 2022-12-14

**Authors:** Ilais Moreno Velásquez, Tobias Pischon

**Affiliations:** 1 Molecular Epidemiology Research Group, Max-Delbrück-Center for Molecular Medicine in the Helmholtz Association (MDC), Robert-Rössle-Straße 10, 13125 Berlin, Germany; 2 Max-Delbrück-Center for Molecular Medicine in the Helmholtz Association (MDC), Biobank Technology Platform, Berlin, Germany; 3 Berlin Institute of Health (BIH) at Charité-Universitätsmedizin Berlin, Core Facility Biobank Berlin, Berlin, Germany; 4 Charité-Universitätsmedizin Berlin, Corporate Member of Freie Universität Berlin and Humboldt-Universität zu Berlin, Berlin, Germany; 5 German Center for Cardiovascular Research (DZHK), Partner Site Berlin, Berlin, Germany

We welcome the initiative by Jordan et al. [1] on distinguishing and proposing uniform definitions for both an exaggerated orthostatic pressor response and orthostatic hypertension. We share the authors’ view that harmonization of guidelines should be encouraged to ensure comparability of results in this research field.

We have, though, some comments regarding the timeframe proposed for the assessments of the upright systolic blood pressure (SBP) measurements. The authors mentioned that SBP while standing should be measured after 1, 3, and 5 min to confirm the persistent character of the blood pressure change. However, they do not provide references to support the time intervals that they propose. While time intervals that are too short may likely not provide reliable results, time intervals that are too long may increase the total time burden of patients or study participants, as well as of study personnel. Although at first sight small differences may seem negligible, when performed daily on a routine basis, this may also increase the financial burden in clinical practice as well as in research settings. Ultimately, these factors may have a negative impact on the acceptability of the measurement, both on the side of the patient (or study participant) as well as on the side of the clinician (or researcher) [2–4]. Therefore, in our opinion, any measurement should be as short as possible, while still providing reliable estimates of upright SBP. We recently investigated, in 522 participants from the pretest of the German National Cohort (NAKO), the feasibility of an extended blood pressure measurement protocol. The protocol included blood pressure measurements three times in the sitting position after 5 min of rest and recorded at 2-min intervals, followed by five measurements upon standing recorded every 45 s [5]. We observed that inclusion of the last measurement did not provide significant further information when classifying study participants according to different blood pressure groups. The Kappa coefficient, which indicates the agreement between inclusion and exclusion of the fifth measurement, was 0.86 [95% confidence interval (CI) 0.81–0.92]. In view of these findings, we prescinded the last measurement, shortened the time interval for blood pressure measurements assessment, and utilized the average of the second to fourth measurement on standing to define standing SBP [5]. Moreover, we found a good reliability for our defined standing SBP in two independent populations within 10 and 34 days of repeated measurements. The intraclass correlation coefficient was 0.70 (95% CI 0.45–0.85) in the NAKO pretest and 0.86 (95% CI 0.70–0.94) in the MetScan study [5]. Thus, we propose to have four measurements to evaluate standing SBP: the first measurement to be assessed after the study participant transitions from sitting to standing, followed by three measurements of blood pressure every 45 s. Further, we consider it important to streamline the proposed guidelines with other ones related to the field. Although substantial heterogeneity still exists between recommendations regarding to timing of blood pressure measurements for the screening of orthostatic hypotension [6], the majority focused on measurements within 1–3 min after standing [7, 8].

In addition, the authors recommended that measurements should be conducted in a quiet surrounding to limit confounding of orthostatic changes by external stimuli and that repeated tested should be conducted on a different day. We would like to add the importance of taking into consideration body, room, and external temperature when assessing the blood pressure parameters, given that they provide important insights on hemodynamic changes in blood pressure [9]. Finally, more research is needed to disentangle whether values lower than the proposed cutoff of 20 mmHg in postural changes in SBP have important clinical implications. Therefore, we propose that analysis using both continuous values and established categorical cutoffs might be of benefit.
